# Developing and Evaluating a Universal Primary School Body Image Program: An Uncontrolled Pilot Study of Butterfly Body Bright

**DOI:** 10.3390/bs16081306

**Published:** 2026-08-01

**Authors:** Stephanie R. Damiano, Hannah K. Jarman, Susan J. Paxton

**Affiliations:** 1 School of Psychology and Public Health, La Trobe University, Melbourne 3086, Australia; susan.paxton@latrobe.edu.au; 2 Butterfly Foundation, Sydney 2065, Australia; 3 School of Psychology, Deakin University, Geelong 3220, Australia; h.jarman@deakin.edu.au

**Keywords:** body image, body dissatisfaction, prevention, children, school, teachers, universal

## Abstract

Body image develops during childhood, yet few universal programs address body dissatisfaction and disordered eating risk across the primary school years. This paper describes the development of Butterfly Body Bright, which is, to our knowledge, a world-first whole-of-school program promoting positive body image and healthy relationships with eating and physical activity across all primary school years. We also describe an uncontrolled pilot study that evaluated the feasibility, acceptability, and impact of components of the program, namely, online staff training and teacher-led lessons from three program themes. Lessons targeted appearance-based teasing, media literacy, body gratitude, and self-compassion. Participants were 12 teachers and 114 Grade 4 to 6 children in Australia, who received one of seven Body Bright lessons. Significant pre- to immediate post-program improvements emerged in state body satisfaction, body appreciation, confidence managing appearance-based teasing, and help-seeking intentions. Specific lessons improved media literacy and appearance comparison and, at a 3-to-7-week follow-up, trait body appreciation. Boys showed improvement in state body satisfaction, confidence managing appearance-based teasing, and appreciation of body functionality. Girls showed improvement in state body satisfaction and media literacy. Butterfly Body Bright showed some promising preliminary results, with small to large effects, and was deemed feasible and acceptable by teachers, supporting the need for a randomized controlled trial.

## 1. Introduction

Childhood is a formative stage for body image (Paxton & Damiano, 2017), which is a broad term that describes an individual’s thoughts, feelings, perceptions, and behaviors towards their body (Thompson et al., 1999). Research with very young children tends to show general satisfaction with their body size (e.g., Damiano et al., 2015). However, body dissatisfaction appears to be increasingly prevalent as children enter their primary (elementary) school years. For instance, research shows that more than half of 6-to-9-year-old girls have reported a desire to be thinner (Slater & Tiggemann, 2016), while around a third of 6-year-old boys have desired a more muscular body (McLean et al., 2018). There is evidence for stability in children’s body image. For example, children who report greater body dissatisfaction have been shown to report continued body dissatisfaction, while those who report greater body satisfaction continue that trajectory throughout childhood and into early adolescence (e.g., Damiano et al., 2019; Lacroix et al., 2022). At the same time, there is evidence for an increase in appearance anxieties with age (e.g., Zimmer-Gembeck et al., 2018). Such findings underscore the importance of establishing a positive foundation for body satisfaction during childhood.

Body dissatisfaction is a key modifiable risk factor for the development of several negative physical, mental, and social issues. For children, an Australian study revealed that greater body concerns in girls at ages 5 and 7 predicted greater dietary restriction by the age of 9 (Davison et al., 2003). In addition, a longitudinal study in the UK demonstrated the concurrent development of body dissatisfaction and eating disorder symptoms between ages 7 and 12 (Evans et al., 2017). Greater body dissatisfaction has also been shown to predict greater declines in academic engagement in third to fifth graders in the US over an 8-week period (Guimond & Laursen, 2022). During adolescence and adulthood, body dissatisfaction is a well-established risk factor for depressive symptoms and episodes (e.g., McLean et al., 2022), onset of smoking (e.g., Kaufman & Augustson, 2008), unsafe sexual behaviors (e.g., Schooler, 2013), disordered eating behaviors, including dietary restriction and unsafe muscle-building behaviors (e.g., Neumark-Sztainer et al., 2006; Sagoe et al., 2014), and clinical eating disorders (e.g., Stice et al., 2011). Importantly, a global study demonstrated that more than one in five children and adolescents have engaged in disordered eating (López-Gil et al., 2023). As a result, the need to prevent the development of body dissatisfaction during childhood has never been stronger.

To achieve this prevention goal, it is critical to mitigate the risk factors and foster the protective factors of body image. Research shows that individual factors, such as self-esteem (e.g., Wojtowicz & von Ranson, 2012) and body appreciation (e.g., Craddock et al., 2025), and sociocultural factors, such as appearance-based teasing (e.g., Zimmer-Gembeck et al., 2018), other peer influences (e.g., Tatangelo & Ricciardelli, 2013), families (e.g., Rodgers et al., 2020, 2024a), and media (e.g., De Coen et al., 2021), are key factors that can influence a child’s body image. In recent research, the negative impact of social media, particularly photo-based social media (e.g., Lonergan et al., 2020), on body image and the need to mitigate these risks has become an increasing focus in young people (e.g., Mazzeo et al., 2024; Rodgers et al., 2024b). Although most social media platforms specify a minimum age of 13 for account holders, a recent study highlights that a substantial proportion of Australian pre-adolescents already hold social media accounts, with social media users reporting greater body image concerns (Fardouly et al., 2020). However, daily average use of social media was revealed as less than half an hour per day (Fardouly et al., 2020). Given this limited engagement with social media among primary school-aged children, alongside strong evidence for the influence of traditional media on children’s body image (e.g., De Coen et al., 2024) and the greater relevance of traditional media formats within the school environment, the focus in the present study is to build foundational, developmentally appropriate media literacy skills across media sources more broadly (including books, television, film, and online content), rather than focusing specifically on social media.

Importantly, a number of individual and sociocultural influences of body image are present within the school environment; hence, the development of many school-based prevention programs. Schools have long been recognized as ideal avenues for the widespread dissemination of universal body image programs (e.g., Yager & O’Dea, 2015). Yet teachers have reported a lack of resources and confidence to talk to children about body image and have identified a need for professional development and age-appropriate resources (Damiano et al., 2018; Pursey et al., 2022). Despite this, teacher-delivered body image and eating disorder prevention programs have demonstrated feasibility and acceptability and shown to positively impact students in primary (e.g., Damiano et al., 2018) and secondary schools (e.g., Wilksch, 2015). Recent meta-analyses have been conducted on existing primary school programs that have addressed several modifiable risk factors (e.g., media literacy and peer teasing/relationships) and protective factors (e.g., celebration of diversity, body acceptance, and positive eating behaviors) with the ultimate goal of preventing body dissatisfaction and eating disorder risk in children (Chua et al., 2020; Pursey et al., 2022). Overall, interventions have been shown to improve body image and related factors such as self-esteem and internalization of appearance ideals (e.g., Chua et al., 2020; Pursey et al., 2022). Despite general improvements to body image from school-based programs, the impact is often short in duration (Berry et al., 2025; Kusina & Exline, 2019). This highlights the need for schools to commit to long-term sustainable approaches.

The importance of cultural and systemic change has been recognized beyond upskilling individuals to be resilient to the sociocultural pressures about appearance and body size and shape (Bell et al., 2017). Schools offer a unique opportunity to consider influences within the broader environment, such as at a policy level (Puhl et al., 2014). Further, through their Health-Promoting Schools framework, the World Health Organization (2021) emphasizes that the health and wellbeing of children can be improved with coordinated and extensive efforts throughout the school community and environment, including curriculum and teaching, school ethos and environment, and partnerships with families and the community. It is further recognized that schools are a dynamic and complex ecosystem and that by involving the whole school community, more supportive and inclusive environments can be fostered (Zhou et al., 2025).

Universal programs have been developed and evaluated that contain curriculum content across the primary school years (e.g., Halliwell et al., 2016) or offer curriculum content and support for teachers and parents (McVey et al., 2007). However, to our knowledge, Butterfly Body Bright is the first primary school body dissatisfaction and eating disorder risk reduction program that adopts a whole-of-school approach, which contains content for school policy and culture, staff training, curriculum content across all primary school years, and resources for families. By applying principles of effective implementation and sociocultural and ecological theories, the aim of Butterfly Body Bright is to prevent the onset of significant body dissatisfaction and later eating disorders in primary school children (aged 5 to 12) by fostering a school culture and environment that support the positive development of body image and eating and physical activity attitudes and behaviors.

This paper outlines the development of Butterfly Body Bright, followed by a small uncontrolled pilot evaluation that assessed components of the program for feasibility, acceptability, and impact. The aims of the pilot study were to determine the feasibility and acceptability of the online staff training and teacher-led lessons and assess the impact on the body image and related variables of children in the upper years of primary school (Grades 4 to 6) who receive one Body Bright lesson. It was hypothesized that children would show pre- to post-intervention improvements in state measures for body satisfaction, confidence to deal with appearance-based teasing, appreciation of body functionality, intention for help-seeking, and media literacy, and a reduction in appearance comparison after receiving one teacher-delivered Body Bright lesson. It was further hypothesized that receiving one Body Bright lesson would improve the trait body appreciation of children at follow-up.

In addition, exploratory analyses were conducted to examine changes in outcomes separately by gender and lesson type. Although the lessons being assessed have content-specific learning objectives (see Section 2.1.3), each lesson tends to incorporate numerous risk and protective factors for children’s body image. Thus, no specific hypotheses were posed for differences between lessons in child outcomes. Further, the existing literature has not clearly identified consistent gender differences in body image or eating outcomes from school-based programs (e.g., Ahuja et al., 2025); thus, gender comparisons were also exploratory.

## 2. Materials and Methods

### 2.1. Intervention

#### 2.1.1. Development of Butterfly Body Bright

Butterfly Body Bright is a program developed and disseminated by Butterfly Foundation, a leading charity organization in Australia that supports the community with body image issues and eating disorders. The development of Butterfly Body Bright as a whole-of-school program (which includes school culture guidelines, staff training, lesson plans, and family resources) was guided by three main activities: (1) a review of the scientific literature and existing resources; (2) advisory and reference groups; and (3) a school strategies study.

Scoping of existing scientific literature and existing resources highlighted the need for the program and identified program components. Sociocultural theories of the development of body image have highlighted the key risk and protective factors that the program should address, including peer, media, and family influences (e.g., Paxton & Damiano, 2017), which are modifiable within the school setting. The program’s development also aligns with principles of effective implementation that highlight the need for long-term and sustainable universal programs for children (Jarman, 2026; Levine & McVey, 2012; Levine & Smolak, 2009).

Next, three groups were formed to guide the development of Butterfly Body Bright. The expert advisory group comprised 12 members with body image, eating disorder, mental health, and/or education expertise. The educator reference group comprised 12 members who were Australian primary school principals, classroom teachers, or wellbeing staff. The parent reference group comprised nine members who were parents of a primary school child or of an adolescent or young adult with lived experience of an eating disorder. The groups contributed to and provided feedback on program design and content. The groups met twice over a two-year period in formal virtual meetings to discuss program aims, scope, and content, whereby they had the opportunity to share ideas and feedback in real-time. All groups were also invited to provide review and written feedback on certain program components as they were completed over the two-year period. Specifically, the expert advisory group provided feedback on all program components; the educator reference group provided feedback on the school culture guidelines, staff training, and lesson plans; and the parent reference group provided feedback on the family resources. The literature review and advisory and reference groups informed the development of the staff training, lesson plans, and family resources.

A school strategies study was then conducted to identify the most important and feasible strategies that primary schools can implement to create a school environment that fosters positive body attitudes and behaviors in Australian children by assessing the support from content experts and educators. The method was modeled on previous policy development in the USA (Puhl et al., 2014). Fifty-three content experts participated (independent of the advisory group), of whom 36% were researchers, 43% were clinicians/counsellors, and 21% were both. The majority (62%) were from Australia, while 28% were from the USA and 10% from elsewhere. Seventy-four Australian educators also participated, of whom 50% were classroom teachers, 21% were the school or assistant principal, 11% were wellbeing staff, 11% were in an administrative or other role, and 7% were physical education teachers.

Participants completed an online survey that invited their support for 31 strategies (derived from the existing literature and agreed on by the project’s expert advisory group) by responding to a 5-point scale from ‘definitely oppose’ to ‘definitely support’. A high level of support was classified for strategies when 90% or more of both the content expert and educator groups supported the strategy. By this standard, a high level of support was achieved for 20 strategies (see Table A1 for a complete list of strategies and their endorsement). The strategy with the highest agreement of support from both groups was that ‘primary schools should aim to ensure the school’s physical environment is accessible to, and inclusive of, community members (staff, students, and families) of diverse body sizes and shapes’ (100% of experts and 98.6% of educators). Open-ended feedback highlighted that the use of the term body acceptance was more appropriate than body positivity in strategies.

**Figure A1:**
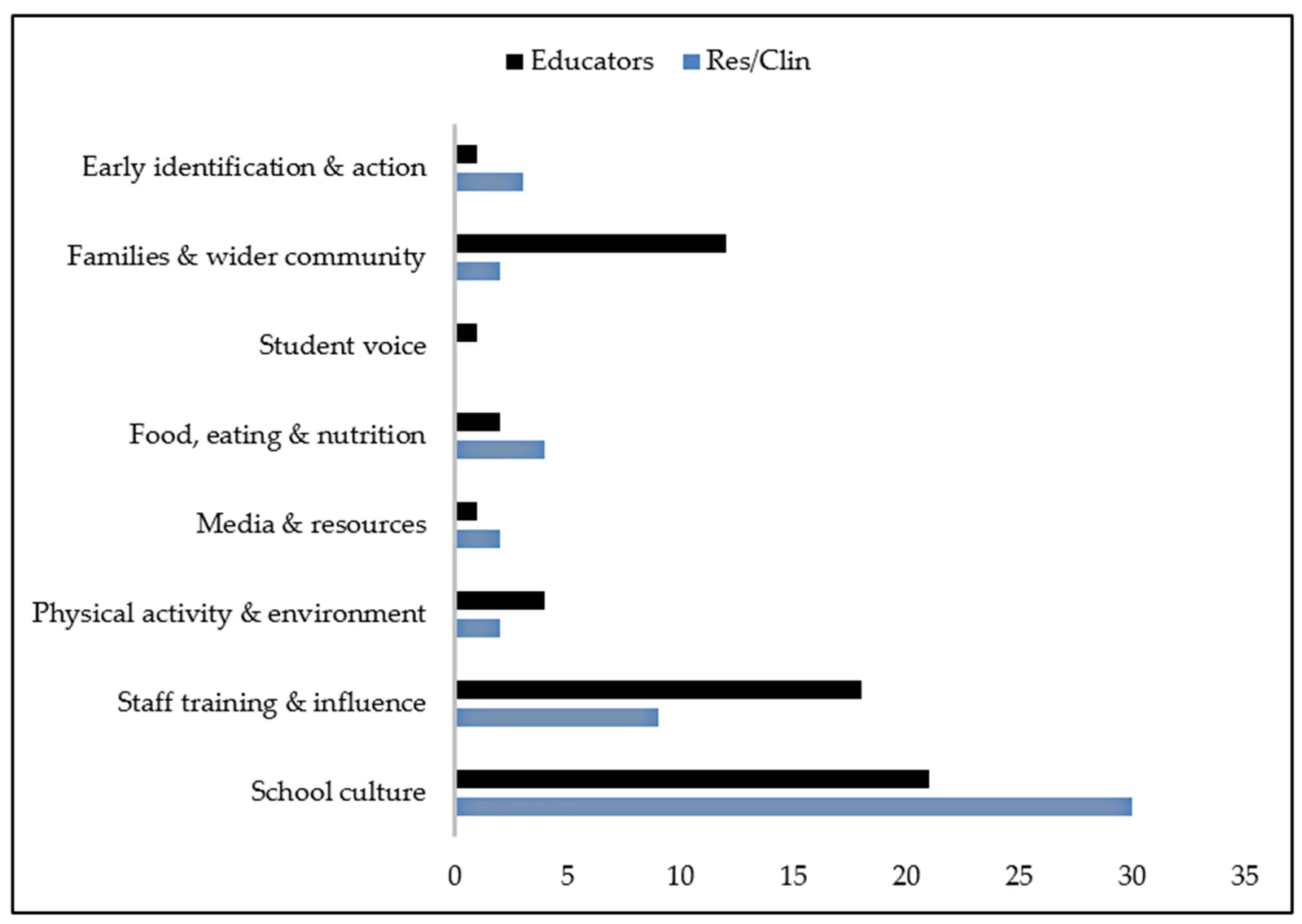
Frequency of the area ranked first as likely to have the most positive impact on children’s body image.

**Figure A2:**
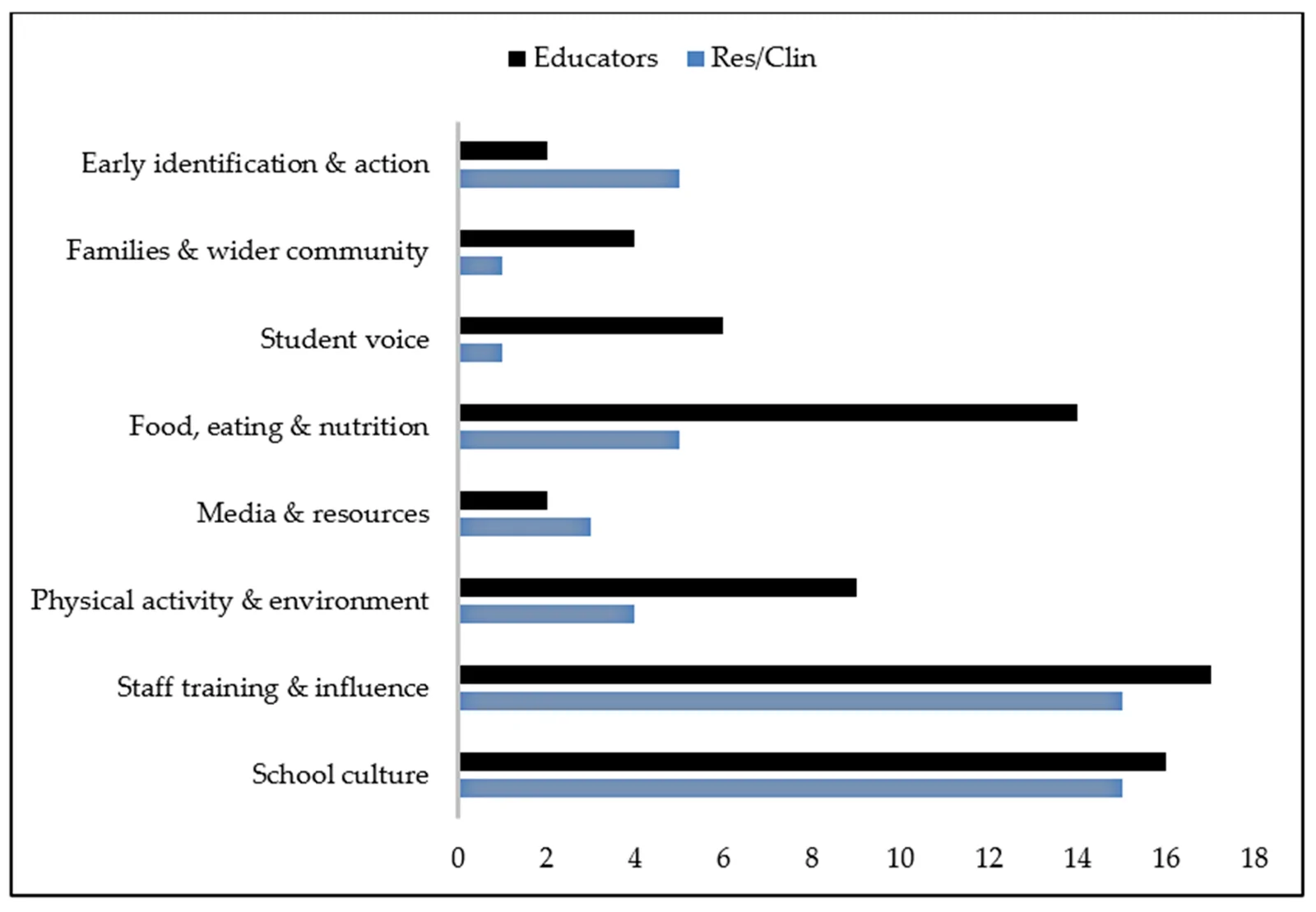
Frequency of the area ranked first for most feasible to implement.

The 31 strategies were also categorized into eight key areas of influence on children’s body image, and participants were invited to rank the top five areas that they rated as most feasible to implement in a primary school and the five areas they rated to be most likely to have the most positive impact on body image in a primary school (see Figure A1 and Figure A2 for the ranked areas). School culture was ranked by both groups as the most likely to have a positive impact on student body image, while staff training and influence was ranked as the most feasible to implement. These strategies helped to inform the development of the school culture and environment guidelines (one of four key program components) in Butterfly Body Bright and levels of engagement for program participation.

#### 2.1.2. Description of Butterfly Body Bright

Butterfly Body Bright is a universal prevention program for primary schools that allows for a whole-of-school approach to foster positive body image and healthy relationships with eating and physical activity in an appearance-inclusive environment. The aim of the school-led program is to empower and educate schools and families in supporting body image through its formative years in childhood. There are four central components to the whole-of-school approach. First, school guidelines that comprise recommendations for improving school culture and environment. These are presented as actions schools can implement (e.g., having a policy for appearance-based teasing), which were derived from the strategies outlined in Appendix A.1. Second, self-paced eLearning for staff (approximately four hours), which supports staff to understand the risk and protective factors for children’s body image and eating and physical activity attitudes and behaviors, the importance of being a Body Bright role model and colleague, early warning signs and intervention, and program implementation. Staff also have available an optional self-reflection module to support their own body appreciation and self-compassion. Third, resources for families that include information sheets and activities that support families in establishing a Body Bright home environment. Schools are also provided with newsletter content to promote Body Bright family resources, and access to a parent seminar. Fourth, 50 teacher-delivered lesson plans across all primary school years (i.e., 5–8 lessons per year from Foundation to Grade 6) that are mapped onto the Australian health and physical education curricula and address some of the modifiable risk and protective factors of body image and attitudes and behaviors toward the body, eating, and physical activity. The lessons incorporate strength- and evidence-based strategies. Central to the program are six themes that map to the word bright, and address numerous influencing factors. Broadly, *Brave* targets peer relationships and appearance-based teasing; *Resilient* promotes media literacy; *Inclusive* targets weight stigma by celebrating diversity; *Grateful* promotes body appreciation and self-compassion and aims to mitigate appearance comparisons in the later years; *Happy* promotes a healthy relationship with physical activity and body functionality; and *Thoughtful* promotes a healthy relationship with eating and food. An ambition of the program is for schools to embed Butterfly Body Bright into their annual activities to provide children with ongoing and repeated learning and support that advances through their primary school years.

#### 2.1.3. Current Study

This study was designed as an uncontrolled pilot evaluation to establish the acceptability, feasibility, and preliminary evidence for program impact to justify and inform the design of a subsequent, adequately powered randomized controlled trial. This is an acceptable first stage in a staged approach to intervention development and evaluation (e.g., Skivington et al., 2021). The pilot study evaluated the staff training and seven Grade 4 to 6 lesson plans. As such, the 12 participating teachers completed the eLearning Body Bright staff training. Due to the pilot nature of this study, three lesson topics were included, namely, Brave, Resilient, and Grateful. In Australia, the curriculum includes the same content descriptors for the two upper years of primary school (i.e., Grade 5 and 6); thus, the Brave and Resilient lessons were the same for both conditions for Grade 5 and 6 classes. Notably, within each lesson topic the same risk and/or protective factors are addressed, but in the most age-appropriate way for that cohort. Table 1 outlines the lessons assessed in the current study.

### 2.2. Ethical Considerations

Approval to conduct this pilot evaluation was granted by the La Trobe University Human Ethics Committee (HEC20466). School principals provided informed consent and signed a research agreement for their school’s participation. Participating classroom teachers signed a research agreement for the completion of staff training and lesson delivery and provided informed consent for survey completion. Parents provided informed consent for their child’s participation in data collection surveys, and children provided assent by completing surveys. All parents were invited to complete the consent form and indicate yes or no to their child’s participation. Of 339 eligible children, 137 (40.4%) parents completed the consent form, in which 127 (92.7%) parents responded yes to their child’s participation in surveys and 10 (7.3%) responded no. All research agreements and consent forms were completed through QuestionPro software.

### 2.3. Procedure

Independent schools in Melbourne, Australia, were invited to participate via email invitation to the principal through existing researcher and Butterfly Foundation networks and databases. Upon principal consent, teachers from Grades 4 to 6, and their students, were invited to participate via email. Teachers completed the online Body Bright staff training prior to accessing lesson content. Classes were randomly assigned to receive one of three Body Bright lessons (Brave, Resilient, or Grateful), and teachers were provided with the relevant lesson plan. The Brave lesson was delivered to two Grade 4, one Grade 5, and one Grade 6 class (*n* = 38 children); the Resilient lesson was delivered to one Grade 4, two Grade 5, and one Grade 6 class (*n* = 43 children); and the Grateful lesson was delivered to one Grade 4, one Grade 5, and two Grade 6 classes (*n* = 33 children). Lessons were delivered by the classroom teacher during regular classroom time determined by the teacher.

Teachers completed one survey following their training and pre-lesson delivery and one post-lesson survey online using QuestionPro software. Teachers did not receive compensation for their participation in the research. Children completed paper surveys immediately pre-lesson, immediately post-lesson, and a 3-to-7-week follow-up. The time variation in the follow-up survey was impacted by various COVID-related lockdowns in Victoria, which impacted school attendance.

### 2.4. Participants

Twelve Grade 4 to 6 classes from two co-educational independent primary schools in Melbourne, Australia, participated in the research. Of the 127 children who had parent consent to participate, 114 completed the pre-intervention survey (Time 1), 113 completed the post-intervention survey (Time 2), and 111 completed the follow-up survey (Time 3). Non-responders at Time 1 were due to absence from school that day or not being in the classroom due to external music lessons. Of the Time 1 participating children, 48.8% were female, 50.4% male, and 0.8% were identified as other, as indicated by the consenting parent. Children were aged between 9 and 13 years (*M* = 10.96, *SD* = 0.84). Twelve Grade 4 to 6 classroom teachers (58.3% female and 41.7% male), who had worked as a primary school teacher between 6 and 23 years (*M* = 10.73, *SD* = 5.12), participated in the research. Ten staff provided their age, which ranged between 28 and 40 years (*M* = 33.80, *SD* = 3.39). Of the 12 classes, four were Grade 4 (four teachers; 32 children), four were Grade 5 (four teachers; 42 children), and four were Grade 6 (four teachers; 40 children).

### 2.5. Materials

#### 2.5.1. Demographic Information

Parents provided their child’s date of birth, from which age was calculated, and gender when completing consent forms. Staff provided their age, gender, and ethnicity in their first survey.

#### 2.5.2. Child Outcomes

Several state measures were completed by children in pre-intervention and post-intervention surveys.

**State body satisfaction** was measured using two questions adapted from recent body image research with children (Matheson et al., 2020). The items were: (1) “How happy do you feel about your body shape, right now”, and (2) “How happy do you feel about the way you look, right now”. The 0-to-10-point response scale was altered from ‘extreme dissatisfaction-extreme satisfaction’ to ‘extremely unhappy-extremely happy’ on a visual analogue scale (VAS). The slight change in language was deemed more likely to be understood by Australian pre-adolescents. A sad face and smiley face emoji were added at the 0 and 10 anchor points to convey directionality. Scores across the two items were combined to provide a mean body satisfaction score, with higher scores indicating greater body satisfaction. VAS has been widely used, particularly among children, to measure body image and found to be valid and reliable (Durkin & Paxton, 2002).

**Confidence to deal with appearance-based teasing** was measured by a single item, “I am confident that I could deal with teasing and bullying about the way someone looks if I saw it happen”, adapted from measures used by Matheson et al. (2020). Children responded on a 5-point response scale from ‘definitely not’ (1) to ‘definitely’ (4), with a middle ‘I don’t know’ (0) option, with higher scores indicating greater confidence.

**Appreciation of body functionality** was measured by a single item adapted from the same research (Matheson et al., 2020) and in the same manner as state body satisfaction. Children were asked, “How happy do you feel about what your body can do, right now?”, with responses on a 0-to-10-point VAS ranging from ‘extremely unhappy’ to ‘extremely happy’ with accompanying emojis as above. Higher scores indicated greater appreciation of body functionality.

**Appearance comparison** was measured by a single item, “I will try not to compare the way that I look with other people”, which was generated for the present study but is broadly reflective of a positively worded version of items from the Physical Appearance Comparisons Scale (Schaefer & Thompson, 2014) items. Children responded on a 5-point response scale from ‘definitely not’ (1) to ‘definitely’ (4), with a middle ‘I don’t know’ (0) option, with higher scores indicating less comparison.

**Intention for help-seeking** was measured by a single item, “When I notice that I am having a hard time or feeling down, I will talk to someone about it”, adapted from established trait measures of self-compassion for adults (Neff, 2003), but simplified for children. Children responded on a 5-point response scale from ‘definitely not’ (1) to ‘definitely’ (4), with a middle ‘I don’t know’ (0) option, with higher scores indicating greater intention to seek help if having a hard time.

**Media literacy** was measured by two items that were slightly adapted from the appearance focus scale of the Critical Thinking about Media Messages measure (McLean et al., 2016). Children were asked, “When I look at ads, I will think about whether the picture has been edited” and “When I look at ads, I will think about what the people who made the ad want me to believe?” and responded on a 5-point response scale from ‘definitely not’ (1) to ‘definitely’ (4), with a middle ‘I don’t know’ (0) option. The mean score of the two items was calculated, with higher scores indicating greater media literacy.

**Trait body appreciation** was assessed at pre-intervention and follow-up using the Body Appreciation Scale-2 for Children (BAS-2C), a 10-item scale that includes statements such as “I feel good about my body” (Halliwell et al., 2017). The original 5-point response scale was slightly adapted to remove ‘rarely’ and instead was presented as a 4-point response scale from ‘never’ (1) to ‘always’ (4). Mean scores were calculated, with higher scores indicating greater body appreciation. The BAS-2C has been found to be understood, valid, and reliable by 9-to-11-year-olds, demonstrating good internal consistency, construct, criterion-related, and incremental validity, as well as stability over time (Jarman et al., 2025). Good internal consistency was also found in the present study (Cronbach’s α = .90 (Time 1); α = .92 (Time 3)).

**Distractor items** were also incorporated into the pre-intervention survey to distract from body-related content, including how happy they feel when playing with friends, their favorite media to watch, things they would like to do more of at school, and their favorite place to go on the weekend.

#### 2.5.3. Teacher Feedback

Teachers responded to numerous questions designed specifically for this study that provided feedback on the online staff training, lessons, and program overall. Feedback on staff training was measured by three items (outlined in Table 5) that were responded to on a 5-point Likert scale from ‘strongly agree’ to ‘strongly disagree’.

Teachers were also asked to respond to two items that assessed their perceived impact of the staff training on their intention to role model positive body image and avoid talking about bodily appearance in a negative way in front of their students. Responses were on a 5-point scale from ‘definitely hindered’ to ‘definitely improved’.

Lesson feedback was provided by indicating the perceived value of lesson content for their students (3-point scale from ‘low’ to ‘high’), whether the lesson content was engaging for students (4-point scale with ‘yes’, ‘somewhat’, ‘no’, and ‘unsure’ response options), and their likelihood of delivering Body Bright lessons from other topic areas to their class in the future (5-point scale from ‘very unlikely’ to ‘very likely’).

Overall program satisfaction was measured by a single statement, ‘I am satisfied with the Butterfly Body Bright program’, and confidence was measured by a single statement, ‘The program content increased my confidence to teach primary school students about body image’. A 5-point Likert scale from ‘strongly disagree’ to ‘strongly agree’ was used.

#### 2.5.4. Lesson Fidelity

Formal lesson fidelity was assessed by authors (SRD & HKJ), whereby one researcher observed a lesson delivery by the classroom teacher. Due to COVID-related restrictions at the time and timetabling challenges, lesson fidelity was observed in three out of 12 lessons. The researchers responded to a series of questions on a 10-point scale about the degree to which lesson goals were met, the rating of the teacher’s adherence to the lesson plan, and students’ lesson activity completion and competence.

Informal lesson fidelity was assessed in the post-intervention survey completed by teachers, whereby teachers indicated how much of the lesson they delivered (4-point scale from ‘all’ to ‘none’) and how easy or difficult it was to stick to the lesson plan (4-point scale from ‘very easy’ to ‘very difficult’). Where a level of difficulty was indicated, an open-text response was invited to provide more information.

### 2.6. Data Analysis

All analyses were conducted using IBM SPSS Statistics (Version 31). Descriptive statistics were calculated for all variables, including means and frequencies. Missing data were observed for several variables at Time 1 and Time 2 (ranging from 1% to 8%), and up to 14% at Time 3. Missing data analyses were conducted using Mann-Whitney U and chi-square tests to compare children with and without missing data. At Time 2, groups only differed on baseline media literacy (*p* = 0.049), with slightly higher scores reported for those with missing data. At Time 3, participants with missing data reported slightly lower baseline body satisfaction (*p* = .023) and appreciation (*p* = .039). No other differences were observed for the outcome variables, nor in relation to age or gender. Overall, these findings suggested limited evidence of systemic attrition. Therefore, analyses were conducted under the assumption that data were missing at random and used available paired observations.

Data were screened for normality using Shapiro–Wilk tests. Analyses revealed that all study variables significantly deviated from a normal distribution (all *p* < .01), suggesting violations of the normality assumption whereby scores were skewed toward positive body image outcomes. Given that outcome variables demonstrated non-normal distributions, Wilcoxon signed-rank tests were used to examine changes in child outcomes from immediate pre-intervention (Time 1) to immediate post-intervention (Time 2), including body satisfaction, appreciation of body functionality, confidence to deal with appearance-based teasing, appearance comparisons, intention for help-seeking, and media literacy. Changes in body appreciation from Time 1 to Time 3 (3-to-7-week follow-up) were also examined using Wilcoxon signed-rank tests. Effect sizes were calculated using *r* = *Z*/√ *n*, with values of 0.10, 0.30, and 0.50 interpreted as small, medium, and large, respectively (Cohen, 1988).

Exploratory subgroup analyses were also conducted to examine intervention effects separately by gender and lesson (Brave, Resilient, Grateful). Due to insufficient sample size, the child whose parent had reported their gender as ‘other’ was excluded from the gender subgroup analysis. Given the small sample sizes and the exploratory nature of these analyses, the results should be interpreted with caution.

## 3. Results

### 3.1. Sample Characteristics

Table 2 presents the descriptive information for child outcome variables pre- and post-intervention and follow-up for the overall sample. At baseline, participants reported high body satisfaction, body appreciation, and appreciation of body functionality, and moderate appearance comparisons, confidence to deal with appearance-based teasing, intention for help-seeking, and media literacy.

Pre-intervention frequencies were calculated. For confidence in dealing with appearance-based teasing, 8.1% of children reported no confidence, while 28.8% reported they don’t know, and 63.0% reported some level of confidence. For trying not to compare their appearance to others, 55.8% of children reported they wouldn’t compare, 23.4% reported they would compare, and 20.7% reported they don’t know. For help-seeking, 33.3% reported they did not intend to seek help if having a hard time, 21.6% reported they don’t know, and 45.0% would seek help. When looking at ads, 30.9% reported they would think about whether the picture has been edited, 37.2% reported they would not, and 31.8% reported they don’t know. When looking at ads, 39.1% reported that they would think about what the people who made the ad want them to believe, 36.3% reported they would not, and 24.5% reported they don’t know.

When reporting how happy they felt about the way they look, their body shape, and the functionality of their body, the majority of children reported a score of 7 or above out of 10, including 82.3% for general appearance, 77.2% for body shape, and 89.4% for body functionality.

### 3.2. Program Impact

From Time 1 to Time 2, children reported significant increases in body satisfaction (*z* = −4.99, *p* < 0.001, *r* = 0.47), with large effects. Small-to-moderate improvements were also found for appreciation of functionality (*z* = −2.53, *p* = .011, *r* = 0.24), confidence to deal with appearance-based teasing (*z* = −2.42, *p* = .016, *r* = 0.23), and intention for help-seeking *(z* = −2.01, *p* = .044, *r* = 0.19). In these latter two, this represented a shift from 63% saying they would feel confident to deal with appearance-based teasing to 70.7%, and from 45.0% intending to seek help to 55.8%. No significant changes were found from Time 1 to Time 2 in media literacy (*z* = −1.89, *p* = .059, *r* = 0.18) or appearance comparisons *(z* = −1.86, *p* = .063, *r* = 0.18), nor for body appreciation from Time 1 to Time 3 (*z* = −1.93, *p* = .054, *r* = 0.19), though results appeared to be approaching significance, with small-to-moderate effect sizes. The proportion of children reporting that they will not compare their appearance to others rose from 55.8% at Time 1 to 69.8% at Time 2.

Table 3 presents means and standard deviations for each outcome variable across timepoints, stratified by gender. Exploratory analyses by gender revealed a significant increase from Time 1 to Time 2 for body satisfaction in both boys (*z* = −3.28, *p* = .001, *r* = 0.44) and girls (*z* = −3.73, *p* <.001, *r* = 0.50), representing large effect sizes. Boys also reported a significant increase in confidence to deal with appearance-based teasing (*z* = −2.99, *p* = .003, *r* = 0.40; medium-to-large effect) and appreciation of body functionality (*z* = −1.97, *p* = .048, *r* = 0.26; medium effect), while girls reported significant improvements in media literacy (*z* = −1.96, *p* = .050, *r* = 0.27; medium effect). No significant changes were observed for boys in appearance comparison (*z* = −1.27, *p* = .204, *r* = 0.17), intention for help-seeking (*z* = −1.27, *p* = .205, *r* = 0.17), or media literacy (*z* = −0.59, *p* = .558, *r* = 0.08). Similarly, no significant changes were observed for girls in confidence to deal with appearance-based teasing (*z* = −0.21, *p* = .832, *r* = 0.03), appreciation of body functionality (*z* = −1.46, *p* = .144, *r* = 0.20), appearance comparison (*z* = −1.36, *p* = .175, *r* = 0.19), or intention for help-seeking (*z* = −1.78, *p* = .076, *r* = 0.24). From Time 1 to Time 3, no significant changes were found in body appreciation for boys (*z* = −1.69, *p* = .091, *r* = 0.24) or girls (*z* = −1.00, *p* = .32, *r* = 0.15) separately.

Table 4 presents means and standard deviations for each outcome variable across timepoints, stratified by lesson. Exploratory analyses by lesson revealed that for the Brave lesson, a significant increase from Time 1 to Time 2 was observed for body satisfaction (*z* = −3.85, *p* < .001, *r* = 0.62), representing a large effect size. Significant increase from Time 1 to Time 3 was also found for body appreciation (*z* = −2.01, *p* = .044, *r* = 0.36; medium effect). No significant changes were observed for confidence in dealing with appearance-based teasing (*z* = −1.95, *p* = .051, *r* = 0.33) or appearance comparison (*z* = −1.95, *p* = .052, *r* = 0.33), although both findings approached significance and demonstrated medium effect sizes. No significant changes were found for appreciation of body functionality (*z* = −1.63, *p* = .104, *r* = 0.26), intention for help-seeking (*z* = −0.96, *p* = .335, *r* = 0.16), or media literacy (*z* = −0.25, *p* = .799, *r* = 0.04).

For the Resilient lesson, significant increases from Time 1 to Time 2 were observed for body satisfaction (*z* = −4.12, *p* < .001, *r* = 0.63), confidence in dealing with appearance-based teasing (*z* = −2.20, *p* = .027, *r* = 0.34), and media literacy (*z* = −2.71, *p* = .007, *r* = 0.41), representing large, medium, and medium-to-large effect sizes, respectively. No significant changes were found for appreciation of body functionality (*z* = −1.23, *p* = .220, *r* = 0.19), appearance comparison (*z* = −1.38, *p* = .167, *r* = 0.22), intention for help-seeking (*z* = −1.05, *p* = .292, *r* = 0.16), or body appreciation from Time 1 to Time 3 (*z* = −1.29, *p* = .196, *r* = 0.20).

For the Grateful lesson, no significant changes were observed across any outcomes from Time 1 to Time 2, including body satisfaction (*z* = −0.07, *p* = .946, *r* = 0.01), confidence to deal with appearance-based teasing (*z* = −0.14, *p* = .892, *r* = 0.02), appreciation of body functionality (*z* = −1.63, *p* = .103, *r* = 0.29), appearance comparison (*z* = −0.48, *p* = .631, *r* = 0.08), intention for help-seeking (*z* = −1.50, *p* = .134, *r* = 0.27), media literacy (*z* = −0.61, *p* = .539, *r* = 0.11), or body appreciation from Time 1 to Time 3 (*z* = −0.09, *p* = .930, *r* = 0.02).

### 3.3. Teacher Feedback Outcomes

Teachers provided feedback following their completion of the staff training, with group data presented in Table 5.

Teachers also self-reported a positive impact on their behaviors, with 75.0% indicating a ‘somewhat’ or ‘definite’ improvement in their intention to role model positive body image in front of their students, and 58.3% indicating a ‘somewhat’ or ‘definite’ improvement in their intention to avoid talking about appearance in a negative way in front of their students.

In the post-intervention survey, teachers reported on lesson feedback, program satisfaction, and confidence. Most teachers rated the perceived value of the lesson content as medium (83.3%), with 8.3% rating it as high and 8.3% rating it as low. Overall, 83.3% of teachers perceived the lesson content as engaging or somewhat engaging for students, and 66.7% of teachers stated it was ‘likely’ they would deliver other Body Bright lessons in the other topic areas in the future, while the remainder were unsure mostly due to curriculum/planning limitations. Overall, 75.0% of teachers were satisfied with Butterfly Body Bright; the remainder were neutral. In addition, 83.3% stated the program included useful information, and 58.3% reported that the program content increased their confidence to teach body image content to primary school students.

### 3.4. Fidelity Outcomes

For researcher-reported fidelity, overall lesson goals were met, and teachers’ adherence to the lesson plans was high, with average scores of 8.67 for both variables across the three lessons. Students’ completion of lesson activities was high, with an average score of 9.00 across the three lessons, and competence of students to complete tasks was also high, averaging 8.33.

For teacher-reported fidelity, 50.0% reported delivering ‘all’ of their assigned lesson, and 50.0% reported delivering ‘most’ of the lesson. Fifty percent of teachers stated it was ‘very easy’ to stick to the lesson plan, while 25.0% stated it was ‘somewhat easy’ and the other 25.0% stated it was ‘somewhat difficult’. The reasons for difficulties included needing to adjust content to meet student needs and engagement or feeling too prescriptive.

## 4. Discussion

Butterfly Body Bright was developed as a whole-of-school body dissatisfaction and eating disorder risk reduction program. This pilot study evaluated the feasibility and acceptability of two key components of the program, namely, the staff training and some teacher-led lessons. In addition, the impact of one Body Bright lesson on Grade 4 to 6 children’s state and trait body image and other key risk and protective factors were assessed. Results showed promising student outcomes, with statistically significant changes and large effect sizes observed in body satisfaction, alongside other variables. Positive teacher feedback demonstrated feasibility, and acceptability of the teacher-led lessons, staff training, and the program overall. Good fidelity was also observed.

As hypothesized, significant changes in children’s body image were reported. Specifically, immediate pre- to post-lesson increases in children’s state body satisfaction were reported with large effects. Significant increases in scores were also revealed for both boys and girls. This is a promising finding and consistent with previous studies that have shown improved body satisfaction in primary school-aged children (Pursey et al., 2021). Exploratory analyses showed significant increases in body satisfaction scores for students who received the Brave and Resilient lessons, but not Grateful. This is an interesting finding that requires future exploration, as the Grateful lesson provides evidence-based strategies to address the protective factors of self-compassion and body gratitude, which support body satisfaction (e.g., Braun et al., 2016; Dunaev et al., 2018). Importantly, these results should be interpreted with caution and require replication with comparison to a control group and larger sample; however, there is preliminary support that Body Bright lessons may foster the state body image of upper primary school children.

Overall, significant pre- to post-intervention increases were observed in children’s confidence in dealing with appearance-based teasing. The change in confidence to deal with appearance-based teasing was also observed for boys, but not for girls. This exploratory finding is consistent with the findings of Matheson et al. (2020) that showed immediate improvement in self-efficacy towards bullying behaviors in 7-to-14-year-old boys and 7-to-10-year-old girls, but not girls aged 11 to 14, following a body image micro-intervention. Importantly, the current study did not assess children’s experience of appearance-based teasing, which may be important to explore in future research. Interestingly, when exploring differences between lessons, children who received the Resilient lesson (i.e., media literacy focused) showed a significant increase in scores measuring confidence to deal with appearance-based teasing, but approached significance (with a medium effect size) for children who received the Brave lesson, which is the lesson that more explicitly addresses appearance-based teasing. This finding will require further examination in a larger sample and with comparison to a control group.

Appreciation of body functionality significantly changed from pre- to post-intervention for the sample overall and for boys. This finding may be consistent with the long-existing notion that for boys, body image is often more about the functional aspects of the body and what it can do and less about aesthetics (Ricciardelli, 2012).

The proportion of children who reported that they would not compare their appearance to others rose by 14% from pre- to post-intervention; however, this change only approached significance for the sample overall. No significant changes were observed in appearance comparison across genders or lessons. These findings are somewhat inconsistent with some research that revealed improvements in appearance comparison for boys and girls (e.g., Bird et al., 2013). However, inconsistency in outcome effects among intervention studies is common in school-based body dissatisfaction and eating disorder risk reduction programs (e.g., Berry et al., 2025).

Notably, intention to seek help or support if they were having a hard time significantly changed from pre- to post-intervention. No differences were observed for genders or lessons. It is encouraging that more children reported an intention for help-seeking following a Body Bright lesson, as this is an important first step to early intervention, and the rate of help-seeking among young people with an eating disorder is concerningly low (e.g., Nicula et al., 2022).

Interestingly, for the sample overall, there was no change in media literacy. Encouragingly, when we examined media literacy in the children who received the Resilient lesson (i.e., the media literacy focus), we saw significant change, which may provide some preliminary support for the media literacy content in Butterfly Body Bright improving children’s media literacy; however, replication with a control group is required. Girls also reported significant change in media literacy. Notably, the sample mean for boys’ media literacy was higher than that of girls; thus, it is possible that boys in this study were already more aware of how images and content are manipulated in media.

Changes in body appreciation at follow-up approached significance, providing partial support for the hypothesis. Nor were significant changes observed for the body appreciation of boys or girls separately. However, it was encouraging to see a significant, and medium effect, change in body appreciation from pre-intervention to follow-up for students who received the Brave lesson. Within the program, the Brave lesson is the same across all year levels, with minor age-relevant adaptations, and is the recommended starting point for classroom delivery of Butterfly Body Bright. Thus, if schools are limited by a packed curriculum (Pursey et al., 2022), it may be relevant to consider whether one lesson may improve trait body image. However, replication with a control group and longer-term follow-up is required.

When interpreting the current findings, it is relevant to note that children in the present study reported generally favorable scores across the majority of body image-related risk and protective factors at baseline, which is consistent with previous research (e.g., Matheson et al., 2020). This may have resulted in ceiling effects, whereby there was limited room for measurable improvement, possibly contributing to several non-significant findings across the outcome variables. Despite this, it was promising to find some significant changes in body image with medium-to-large effect sizes. In addition, the subgroup analyses involved substantially smaller sample sizes after separating by gender and intervention lesson, reducing statistical power to detect meaningful differences. Consequently, these subgroup findings should be interpreted as exploratory rather than as evidence of differential intervention effectiveness and replicated with larger samples.

Regarding the feasibility and acceptability of Butterfly Body Bright, teachers reported a high level of satisfaction with the program and valued the program for students. All teachers reported that the Body Bright staff training explained important concepts well, and most teachers liked the online format of the training and felt more confident to teach about body image. Positive self-reported impacts on teachers’ behavioral intentions were also apparent. Importantly, as with all self-report assessments, social desirability in responding should be considered when interpreting these results. Despite previous research highlighting the needs of teachers to have more professional development on children’s body image (Damiano et al., 2018; Pursey et al., 2022), around 42% reported that the staff training filled an important gap in their professional development, though 50% remained neutral. Fidelity in the lesson was also high for the small number of lessons observed, which overall aligned with the teachers’ self-reporting in those three lessons. Unfortunately, formal fidelity observations for all classes were restricted by practical constraints on researchers having limited access to schools at the time of data collection, largely due to COVID-related restrictions on visitors entering school premises and the extensive lockdowns experienced in Victoria. Overall, these findings provide preliminary support for the feasibility and acceptability of the Body Bright staff training and lessons within primary schools.

As with all research, this study must be considered in light of some limitations. Firstly, the absence of a control group limits the examination of the program’s impact on children; thus, these findings should be interpreted with caution and replicated with a control group. Additionally, although the primary analyses focused on a small number of theoretically and empirically supported outcomes, multiple tests were conducted, including exploratory subgroup analyses. Consequently, there is a possibility of Type I error, particularly for the subgroup findings. Further, the follow-up period was short and varied across classes, given our need to be flexible with data collection due to COVID-related lockdowns and school interruptions in Victoria, Australia, at the time. It will be important for future research to attempt to have a more consistent follow-up timeframe. We also only evaluated a small subset of the lessons due to the pilot nature of the study.

Notably, not all child measures have been fully validated in primary school-aged children. This reflects a broader gap in the field, as psychometrically validated measures for the risk and protective factors of body image are limited for children. Given this, and because the current study aimed to assess a broad range of risk and protective factors relevant to the intervention, brevity over comprehensiveness in measurement was prioritized to keep participant burden manageable for the children completing multiple measures within a single classroom session. Several single-item measures were therefore used in place of longer scales. Where possible, these items were drawn from, or adapted from, prior peer-reviewed research conducted independently of the current study (e.g., Matheson et al., 2020), providing some precedent for their use with children in this age range, although this does not constitute formal psychometric validation. We also note that the modification of the BAS-2C response scale from a 5-point to a 4-point scale has not been independently validated, and while informed by broader evidence on response format comprehension in children, future research should examine the psychometric properties of this shortened version directly. There is also limited generalizability of the findings given the sample was from independent schools in a higher socioeconomic area of Melbourne, Australia. These limitations, alongside the promising preliminary results, justify the need for a larger effectiveness trial with a more robust sample size and diversity in school locations.

## 5. Conclusions

Butterfly Body Bright was developed as a whole-of-school program. Evaluation of the staff training and lessons showed feasibility and acceptability by primary school teachers. The current findings also provide preliminary support for the Body Bright lessons being associated with positive change in upper primary school children’s state body satisfaction, appreciation of body functionality, confidence to deal with appearance-based teasing, and intention for help-seeking, with mixed patterns from exploratory analyses for girls and boys. Positive change in children’s media literacy was also observed alongside receiving the Resilient lesson, while longer-term positive change in trait body appreciation was observed among children who received the Brave lesson.

These findings are promising given the moderate-to-large effect sizes, even with the likelihood of ceiling effects. The findings lend support to the program’s wider dissemination as a universal school-led program. The results from this small uncontrolled pilot evaluation were a first step in understanding the acceptability and preliminary impact of Butterfly Body Bright. However, further evaluation is required in an adequately powered randomized controlled trial to replicate findings and better understand the impact of the whole-of-school approach.

## Figures and Tables

**Table 1:** Overview of the Body Bright lessons assessed in the pilot evaluation.

Lesson	Learning Focus	Learning Strategies
Grades 4–6 (Brave) Butterfly Body Bright Promise	To promote inclusion, connection, and a sense of belonging, and to prevent appearance-related negative comments, teasing, and exclusion.	Involve students in a brainstorming activity to develop a class agreement for positive peer interactions across the six Body Bright themes for greater impact and relevance (modeled from Haines et al., 2007).
Grade 4 (Resilient) Spot the difference: Photoshop vs. reality	To promote critical analysis of the way images have been manipulated to discourage students from ‘buying into’ the images they see in media.	Apply media literacy principles of ‘authorship’ and ‘format’ as students explore the fictional nature of media images and the use of manipulation techniques to modify media images (not appearance-focused).
Grade 5/6 (Resilient) Adverts of the future	To encourage students to challenge the appearance ideals they see in media.	Apply the media literacy principles of ‘format’, ‘content’, and ‘purpose’, and create media texts that promote appreciation of body function, rather than appearance.
Grade 4 (Grateful) I love that my body can	To encourage students to develop ways of feeling gratitude towards their body through reflection on their body senses.	Explore body gratitude for the senses and create positive body affirmations.
Grade 5 (Grateful) Body kindness	To encourage students to recognize the impact of appearance comparisons and to develop strategies to overcome comparisons.	Apply cognitive dissonance strategies to mitigate appearance comparison and promote self-acceptance. Practice self-compassion. Create positive affirmations.
Grade 6 (Grateful) Giving thanks for my body	To encourage students to recognize the impact of appearance comparisons and develop strategies to overcome comparisons and body gratitude.	Apply cognitive dissonance strategies to mitigate appearance comparison and cognitive restructuring. Practice body gratitude. Create positive affirmations.

**Table 2:** Descriptive data at pre- and post-intervention and follow-up for child outcome variables.

Variable	Time 1 (*N* = 114)		Time 2 (*N* = 113)		Time 3 (*N* = 111)	
	*M* (*SD*)	Range	*M* (*SD*)	Range	*M* (*SD*)	Range
Body satisfaction	**7.76 (1.74)**	**2.00–10.00**	**8.20 (1.76)**	**0.00–10.00**	-	-
Confidence to deal with appearance teasing	**2.20 (1.51)**	**0.00–4.00**	**2.49 (1.45)**	**0.00–4.00**	-	-
Appreciation of body functionality	**8.25 (1.93)**	**0.00–10.00**	**8.56 (1.71)**	**0.00–10.00**	-	-
Appearance comparison	2.32 (1.44)	0.00–4.00	2.56 (1.34)	0.00–4.00	-	-
Intention for help-seeking	**1.98 (1.27)**	**0.00–4.00**	**2.19 (1.35)**	**0.00–4.00**	-	-
Media literacy	1.84 (1.06)	0.00–4.00	2.07 (1.08)	0.00–4.00	-	-
Body appreciation	3.26 (0.59)	1.78–4.00	-	-	3.34 (0.58)	1.50–4.00

Note: Significant effects are presented in bold.

**Table 3:** Descriptive data at pre- and post-intervention and follow-up for gender-based outcome variables.

	Boys			Girls		
Variable	T1 (*n* = 56)	T2 (*n* = 56)	T3 (*n* = 50)	T1 (*n* = 56)	T2 (*n* = 56)	T3 (*n* = 47)
	*M* (*SD*)	*M* (*SD*)	*M* (*SD*)	*M* (*SD*)	*M* (*SD*)	*M* (*SD*)
Body satisfaction	**8.05 (1.61)**	**8.45 (1.75)**	-	**7.47 (1.85)**	**7.94 (1.78)**	-
Confidence to deal with appearance teasing	**2.09 (1.47)**	**2.64 (1.30)**	-	2.31 (1.56)	2.33 (1.61)	-
Appreciation of body functionality	**8.56 (2.00)**	**8.95 (1.31)**	-	7.96 (1.84)	8.18 (1.98)	-
Appearance comparison	2.27 (1.38)	2.53 (1.27)	-	2.35 (1.52)	2.58 (1.43)	-
Intention for help-seeking	1.87 (1.25)	2.11 (1.33)	-	2.07 (1.30)	2.28 (1.38)	-
Media literacy	2.07 (1.09)	2.20 (1.13)	-	**1.61 (0.98)**	**1.92 (1.00)**	-
Body appreciation	3.34 (0.54)	-	3.48 (0.48)	3.17 (0.64)	-	3.21 (0.64)

Note: T1 = Time 1, T2 = Time 2, T3 = Time 3. Significant effects are presented in bold.

**Table 4:** Descriptive data at pre- and post-intervention and follow-up for lesson-based outcome variables.

Variable	Brave Lesson			Resilient Lesson			Grateful Lesson		
	T1 (*n* = 38)	T2 (*n* = 38)	T3 (*n* = 32)	T1 (*n* = 43)	T2 (*n* = 43)	T3 (*n* = 40)	T1 (*n* = 33)	T2 (*n* = 32)	T3 (*n* = 26)
	*M* (*SD*)	*M* (*SD*)	*M* (*SD*)	*M* (*SD*)	*M* (*SD*)	*M* (*SD*)	*M* (*SD*)	*M* (*SD*)	*M* (*SD*)
Body satisfaction	**7.90 (1.65)**	**8.55 (1.20)**	-	**7.85 (1.61)**	**8.51 (1.37)**	-	7.48 (2.03)	7.34 (2.44)	-
Confidence to deal with appearance teasing	2.23 (1.44)	2.67 (1.37)	-	**2.00 (1.63)**	**2.36 (1.50)**	-	2.42 (1.42)	2.45 (1.50)	-
Appreciation of body functionality	8.11 (2.10)	8.42 (1.90)	-	8.77 (1.36)	9.02 (1.12)	-	7.73 (2.24)	8.13 (2.00)	-
Appearance comparison	2.31 (1.47)	2.86 (1.13)	-	2.33 (1.51)	2.59 (1.32)	-	2.30 (1.36)	2.19 (1.53)	-
Intention for help-seeking	2.11 (1.16)	2.25 (1.46)	-	2.26 (1.26)	2.44 (1.30)	-	1.48 (1.30)	1.78 (1.21)	-
Media literacy	1.75 (1.08)	1.74 (1.16)	-	**1.88 (1.20)**	**2.51 (0.95)**	-	1.89 (0.83)	1.84 (0.94)	-
Body appreciation	**3.18 (0.58)**	**-**	**3.32 (0.60)**	3.37 (0.48)	-	3.44 (0.48)	3.19 (0.74)	-	3.23 (0.68)

Note: T1 = Time 1, T2 = Time 2, T3 = Time 3. Significant effects are presented in bold.

**Table 5:** Teacher feedback on the Body Bright staff training.

Item	% Agree	% Disagree	% Neutral
Important concepts/ideas (e.g., body image, weight bias) were explained well.	100%	-	-
The online training filled an important gap in my professional development.	41.7%	8.3%	50.0%
The online format of the training worked well.	66.7%	8.3%	25.0%

Note: % agree responses combined responses ‘strongly agree’ and ‘mostly agree’. % disagree responses combined responses ‘mostly disagree’ and ‘strongly disagree’.

## Data Availability

As per the ethics approval granted for this project, the data are not publicly accessible or stored.

## Abbreviation

The following abbreviation is used in this manuscript:

VAS	Visual analogue scale

## Appendix A

### Appendix A.1

**Table A1:** Group support for the 31 strategies in the school strategy study.

Strategy	Content Experts (% Support)	Educators (% Support)
**School culture:**		
1. Primary schools should include a statement in the Code of Conduct about providing a body positive environment, one which celebrates diversity in, respect for, and inclusion of all body sizes, shapes and appearance of staff and students.	94.3%	85.1%
2. Primary schools should implement a whole learning community approach to positive body image for staff, students, and families.	94.3%	89.2%
3. Primary schools should demonstrate a wide diversity of body sizes, shapes, and appearance in all public and promotional material and posters.	**96.2%**	**93.2%**
4. Primary schools should adopt zero tolerance to appearance-based teasing and/or bullying (including online and social media) and treat as seriously as any other kind of bullying within the school community. [This may be in addition to existing anti-bullying policies, student Code of Conduct, or embedded in classroom inclusion and engagement norms.]	**96.2%**	**95.6%**
**Staff training and influence:**		
5. Primary schools should aim to provide all staff with professional development on acceptance of appearance diversity, body dissatisfaction and eating disorder prevention, and strategies to facilitate early identification and referral.	**92.5%**	**91.9%**
6. Primary school staff should be encouraged to be role models of positive body image and body attitudes, including acceptance of body diversity.	**92.5%**	**95.9%**
**Physical activity and environment:**		
7. Primary school staff and students should avoid weighing and calculating the Body Mass Index (BMI) of students (including but not limited to for classroom activities, as part of physical education or for the reporting to parents). [Exceptions may include: if a school nurse is required to weigh a child for a justified reason or for health research conducted by government or research organizations. However, measurement should not be done in front of peers, scale results should be disguised in a manner so a child is unaware of their weight (e.g., asking child to step on scales backwards), and not providing feedback on the number to the child.]	94.3%	85.1%
8. As part of health, nutrition, and physical activity education, primary schools should include content and activities that aim to promote positive body image, and protect against negative body image messages and related negative attitudes.	**96.2%**	**97.3%**
9. School-based physical activity should include a non-competitive, non-weight-loss, fun, safe, and secure focus, regardless of a student’s weight, size, or shape.	**92.5%**	**90.5%**
10. Primary schools should aim to ensure the school’s physical environment is accessible to, and inclusive of, community members (staff, students, and families) of diverse body sizes and shapes.	**100%**	**98.6%**
11. Primary schools should aim to ensure that their school uniform is inclusive of all children regardless of their size and shape, gender identity, and cultural norms.	**98.1%**	**98.6%**
**Media and resources:**		
12. Primary school staff should select learning materials and stimulus, including the use of newspapers or magazines for activities, which align with the school’s commitment to celebrating and respecting diversity in appearance.	**92.5%**	**93.2%**
13. Primary schools should audit printed and digital content available to students to determine whether the accessible written and visual content is age appropriate and reflective of body positive messaging and imagery.	84.9%	83.6%
14. Primary schools should provide media literacy programs to educate students to be media literate (including social media literate).	**96.2%**	**93.2%**
**Food, eating, & nutrition:**		
15. Food provided within the school environment (e.g., canteen, school events, prizes) should promote a balance of food options from all food groups.	**94.3%**	**97.3%**
16. Primary school canteens should ensure their marketing does not increase stigma around food and the body (e.g., have one line for all foods rather than a separate line for salads and a line for ‘other/less nutritious foods’).	94.3%	89.0%
17. In nutrition education and school activities, primary schools should aim to promote a positive relationship between the body and food by promoting healthy eating attitudes and behaviors, mindful eating, and eating and exploring all food groups.	**94.3%**	**100%**
18. In nutrition education and school activities, primary schools should aim to avoid promoting negative attitudes towards food (e.g., labelling food as good/bad).	96.2%	86.3%
19. In nutrition education and school activities, primary schools should aim to avoid content or activities that may increase the risk of eating disorders (e.g., teaching about dieting, reading food labels, keeping food diaries).	**94.3%**	**90.4%**
20. Irrespective of a child’s weight, size, or shape, students should not be encouraged to diet or restrict food intake, or compensate (e.g., exercise) following the consumption of certain foods (e.g., low nutrient ‘sometimes’ foods).	**96.2%**	**93.2%**
21. If a staff member is concerned about the contents of a child’s lunchbox, they should have a private conversation with the child’s parents about those concerns, rather than sending a note in the lunchbox.	77.4%	89.0%
22. Primary school staff should avoid, and students should be discouraged from, commenting on and comparing lunchbox contents (whether positive or negative).	90.6%	75.3%
23. Primary school staff should ensure that students are provided with adequate time and space to eat, but should not be responsible for monitoring the consumption of or nutrient contents of food.	81.1%	82.2%
24. Primary schools should aim to provide opportunities for students to positively engage with food (e.g., school garden, cooking activities, celebration of multicultural events with food).	**94.3%**	**100%**
**Student voice:**		
25. Primary schools should provide opportunities for students to create campaigns or projects, or advocate for initiatives and strategies that support positive body image and appearance diversity in their school.	**98.1%**	**97.2%**
**Families and wider community:**		
26. Primary schools should provide families with information to help them to support children to develop positive body image and a healthy relationship with food.	**92.3%**	**93.1%**
27. Primary schools should provide families with information about eating disorders and pathways for additional resources and support.	82.7%	77.8%
28. Primary schools should encourage parents and families to be positive role models by demonstrating accepting attitudes and behaviors towards their own and other’s bodies.	88.2%	87.5%
29. Primary schools should aim to form partnerships with services and organizations who can support them to promote positive body image in their school.	**92.3%**	**95.8%**
**Early identification and action:**		
30. Primary schools should have a protocol for appropriately supporting any student diagnosed with an eating disorder.	**92.3%**	**91.7%**
31. Primary schools should have a protocol for response and appropriate action for when a child is showing concerns about their body image, eating, or weight.	**96.2%**	**94.4%**

Note: A high level of agreement (>90%) across both groups are bolded.
